# Regulation 2017/745 on medical devices, two major innovations: 1) the physiological action of devices consisting of natural materials such as vegetal matrices; 2) the chemical-physical-mechanical action of devices made of “substances”, which as such are artificial derivatives

**DOI:** 10.3389/fdsfr.2024.1389406

**Published:** 2024-04-03

**Authors:** Marcella Marletta

**Affiliations:** ^1^ Past Director of the General Directorate of Medical Devices and Pharmaceutical Services of the Italian Ministry of Health, Rome, Italy; ^2^ General Director of the Academy for Health and Clinical Research, Rome, Italy

**Keywords:** medical devices, medical devices made of substances, medicinal product, substance, natural materials, vegetal matrix, mechanism of action, physiological

## Abstract

The Medical Device Regulation 2017/745 (MDR) enables the development of a wide range of innovative products. With respect to Directive 93/42, the MDR explicitly identifies the so-called “medical devices made of substances” (MDMS) through specific requirements. In addition, the MDR expands the definition of medical device (MD) by including the “modification of a physiological or pathological state” as a medical purpose specific to devices. This clarifies that materials interacting with the human body in such a way as to modify its “state” are medical devices. Natural materials, such as vegetal matrices, are characterized by the presence of both functional and structural interactions between their components; they can thus be described as “network/s" and interact with the human body in a coordinated, complex way. Since the “state” of the human body is a network of biological functions, the “network/s over a network” interaction between the natural material and the human body is likely to modify the “state” of the human body. Thus, therapeutic products consisting of natural materials, such as vegetal matrices, seem to fit perfectly into the definition of a medical device. Here we analyze the main characteristics of medicinal products, of medical devices made of substances and of medical devices consisting of natural materials. We see that medicinal products and medical devices made of substances have the common characteristic of being based on substances, either synthetic or derivatives of natural materials, but differ in their mechanism of action. On the other hand, medical devices constituted of natural materials relate to the general category of medical devices and cannot be characterized by any single component, identified as an active component. We also discuss how these characteristics relate to the mechanism of action of each type of product. This analysis should allow to identify the most appropriate path for each product, a necessary step to promote research and development of innovative therapies for a large number of unmet medical needs.

## 1 Introduction

Medical devices (MDs) and medicinal products (MPs) represent the therapeutic armamentarium available to modern society and must meet the unprecedented and constantly evolving challenges posed by acute and chronic diseases. The legislations regulating MDs and MPs share the objective of ensuring safe and effective products, but they have quite opposite approaches and refer to very different products. The development of the necessary innovative products for human health is possible only within the right legislation; a careful analysis of the approach behind each legislation is presented in this article. Directive 2001/83 on medicinal products (Directive) (European Commission (EC), 2001) refers to the “Old Approach”, while Regulation 2017/745 on medical devices (MDR) (European Commission (EC), 2017) refers to the so-called “New Approach” (European Commission (EC), 1985; European Commission (EC), 2022). The former is product-oriented: the research and development process is established *a priori*, including detailed technical requirements. The development of a product under the “Old Approach” requires not only that it is included in the scope of the legislation, primarily in its definition, but also that it has the characteristics necessary to comply with the legislation.

Directive 2001/83 defines MPs as “any substance or combination of substances” that produces therapeutic effects by modifying physiological functions through a pharmacological, immunological or metabolic (PhIM) mechanism of action (MoA). The MDR, on the other hand, defines MDs as “any instrument, material or other article” that achieves any of the medical purposes described in the definition through a non-PhIM MoA. In contrast to the “Old Approach”, “New Approach” regulations require compliance with the general requirements specific to each piece of legislation, making the manufacturer responsible to comply with the law by the most appropriate means. “New Approach” regulations are therefore inherently predisposed to continually overcome the state of the art.

The definitions of MPs and MDs have no terms in common; rather, their distinctive features are opposite, starting with the nature of the product itself. The main characteristic of MPs is that they are a “substance”, whereas MDs are mostly an object or a “material”. This difference seems to be at the root of the two opposite mechanisms of action. A “substance”, as defined by the Directive, is a “matter” that can have different origins, chemical or natural. It is necessary to analyze in depth the meaning of this term by means of the structural and functional characteristics of MPs described in Annex I to Directive 2001/83 (Annex I). Annex I describes the “Analytical, pharmaco-toxicological and clinical standards and protocols.” to which MPs must conform to be authorized. Since the Directive refers to the “Old Approach”, it seems methodologically sound that a careful analysis of Annex I should point out the particularities of MPs. Thus, we find that each “substance” of the MP must have a specific role (active substance or excipient) and a qualitative chemical identification described by a molecular formula with its mass (Annex I Module 3, 3.2.1.1.). The pharmacodynamics (MoA and effects) and pharmacokinetics, both preclinical (Module 4) and clinical (Module 5), of the active substance are required. The function of each excipient (preservative, stabilizer, etc.) must also be indicated. Each substance must be quantified (Module 3, 3.2.2.1). These characteristics are specific to well-defined individual molecules, whether of synthetic or natural origin. When dealing with complex matter, it seems that reductionist operations such as marker selection are necessary to make the complex matter meet the characteristics of a “substance” (EMA/HMPC/CHMP/CVMP/162241/20051-Rev3). Conceptually, the interaction between the substance and the human body is thus “pinpointed”, meaning it entails targeted “one to one” or “one to many” interactions between an appropriately identified active substance and one or more receptors (known or unknown) linked with the target biological function. It seems entirely consistent that products that cannot be traced back to single isolated molecules should refer to a regulation with an alternative approach to that of the Directive, such as MDR.

An analysis of the MDR seems to indicate that it specifically identifies the two products discussed below, medical devices made of substances and devices made of natural materials, to ensure their development as devices.

## 2 Innovations brought by Regulation 2017/745 with the inclusion of substance-based medical devices

The MDR replaces Directive 93/42 and extends its mandate. A careful reading shows that it precisely opens to products now commonly referred to as “medical devices made of substances” (MDMS). MDMS were included in Directive 93/42, but not specifically described, and were therefore referred to as “borderline” until the MDR was published. They include devices made of chemically defined substances and devices derived from biological entities.

MDMS, although not defined by the MDR, are precisely regulated by specific general safety and performance requirements (GSPRs) listed in Annex I of the MDR (e.g., GSPRs 12.2, 13.3, 23.2(r), 23.4(t)), a classification rule (Rule 21) and a specific certification procedure (Annex IX 5.4). Analysis of these GSPRs shows that MDMS have the same distinguishing feature as MPs. This characteristic is the determinability of the identity, quantity and function of each component, expressed as a well-defined molecule. In fact, MDMS should provide information similar to that characterizing MPs: a list of identified and quantified components, absorption, distribution, metabolism and excretion of at least the active substance, and precise identification of the site of action of the product. In fact, for the evaluation of MDMS, explicit reference is made to the relevant requirements of Annex I of Directive 2001/83. In case of systemic action, the European Medicines Agency (EMA) or the national competent authorities for MPs must comment on the compliance of the product with these requirements.

Where MDMS are derived from chemically definable substances of biological origin (human, animal or other), the MDR adds GSPR 13.3. This requirement refers to those “devices manufactured using non-viable biological substances other than those referred to in 13.1 [of human origin] and 13.2 [of animal origin]. The process of synthesis or isolation of derived substances yields substances with chemical validation, such as glycerol, simethicone, dimethicone, sucralfate, antacids, hyaluronic acid of various viscosities, polysaccharides, alginate, flavonoids, glucomannan, etc., or substances with biological validation, e.g., yeast or bacteria.

The main difference between MDMS and MPs is the mechanism of action. The standard mechanisms of MDMS are mechanical, physical or chemical (Racchi et al., 2016; Sardi et al., 2018).

## 3 Clarification in Regulation 2017/745 of devices modifying a “state” and implications on the mechanism of action of such devices when constituted by vegetal matrices: concept of “physiological action"

Materials such as natural materials appear to be particularly compliant with the MD definition. An example of a natural material is a vegetal matrix. It is characterized by a large number of components interacting within the matrix in a way similar to that in the plant. This is possible when the manufacturing process does not isolate single component molecules by processes that are considered artificial for this reason (thus producing MPs/MDMS of natural origin). The matrix is thus characterized by interactive networks of components. The interactions affect the reactivity of the components, resulting in the so-called “matrix effect” or “emergent properties”. The matrix effect shows that the structural and functional properties of the matrix cannot be attributed based on the properties of the individual isolated components when these are studied in isolation (Lehn, 2002; Yong et al., 2022). Typically, a matrix has self-assembling and self-organizing properties, resulting in supramolecular structures and functional interactions that are able to respond to different environmental conditions (Lehn, 2002). This phenomenon has been specifically attributed to living matter. The networked interactions within a matrix are relevant to the networked interaction between the physiological functions of the human body when they maintain a physiological state or re-establish the physiological state from a pathological state (Stear, 1973; Bartsch, 2015; Ivanov, 2021).

It appears that the MDR has specifically defined these products as devices by specifying that a device “modifies a physiological or pathological process or state”. Compared to Directive 93/42, which limited the device to the modification of pathological processes, the mandate to modify a “state” of the human body seems an invitation to evolve the state of the art. It is also an alternative to MPs which, by definition, modify single biological functions. Thus, it seems that the “network/s over a network” interaction between natural materials and the human body could be considered as characteristic of medical devices. Such interaction of a natural material with the human body differs fundamentally from the “pinpointed” interaction of a substance with its receptor(s) (the PhIM mechanism of MPs) and from the mechanical/chemical/physical mechanisms of MDMS. The network mechanism accompanies, in each specific context, the physiological actions underlying the state in question, in a coordinated, circular, non-linear manner, to be further discussed and investigated.

## 4 Discussion

Today, more than ever, it is necessary to promote innovation, and living matter has always been a source of cures. If the challenge of the first MP directive, Directive 65/65 (EU, 1965), was to regulate “new chemical entities” to prevent tragedies such as that involving thalidomide, the real challenge today is to regulate natural materials. These materials are characterized by the complexity, interconnectedness, structural and functional redundancy inherent in materials that have been suitably processed from living matter to maintain a matrix that provides the “matrix effect”, and such a matrix is considered instead of markers. Natural materials are fundamentally different from “substances”, including substances of natural origin. Since they are not represented by their individual components, they need a dedicated model. Therefore, to describe natural materials it is necessary to extend the reductionist approach and use the innovations of the last century. Conceptually, this means referring to systems theory. From an experimental point of view, preclinical evidence involves systems biology approaches such as omics sciences (e.g., genomics) and bioinformatics evaluations.

These allow appropriate assessments of the matrix (the acting network/s), the human body (the receiving network) and allow to consider the interaction between the two as a “network/s over a network” interaction. A mechanism that accompanies, in each specific context, the coordinated redundancy and resilience that characterize physiology could be called a ‘physiological mechanism of action’ and could be characterized by a network paradigm, distinct from the targeted and non-targeted models that describe the PhIM and the mechanical/chemical/physical mechanism, respectively. The scientific evidence made possible by current technological advances is beginning to describe this paradigm. The question then seems to be: is there an existing regulatory framework that allows research and development of products characterized by this paradigm? The pharmaceutical approach, rigid and reductionist, does not meet the needs of complexity and therefore fails to introduce the sciences of living matter into medicine. See in this respect the inability of Directive 2001/83 to promote the development of innovative therapeutic products that do not consist of chemically and functionally determinable substances. For example, to adhere to the pharmaceutical “pinpointed” model, the complexity of vegetal materials must be reduced to specific active substances or markers. Otherwise, important exemptions from compliance with Annex I requirements, such as pharmacodynamics and pharmacokinetics, will be required, as is the case for traditional herbal medicinal products. The regulatory framework for traditional herbal medicines, Directive 2004/24 (EU, 2004), is also inappropriate, as the marketing of these products is based solely on long-standing use, and does not allow for the regulation of innovative vegetal matrices.

**FIGURE 1:**
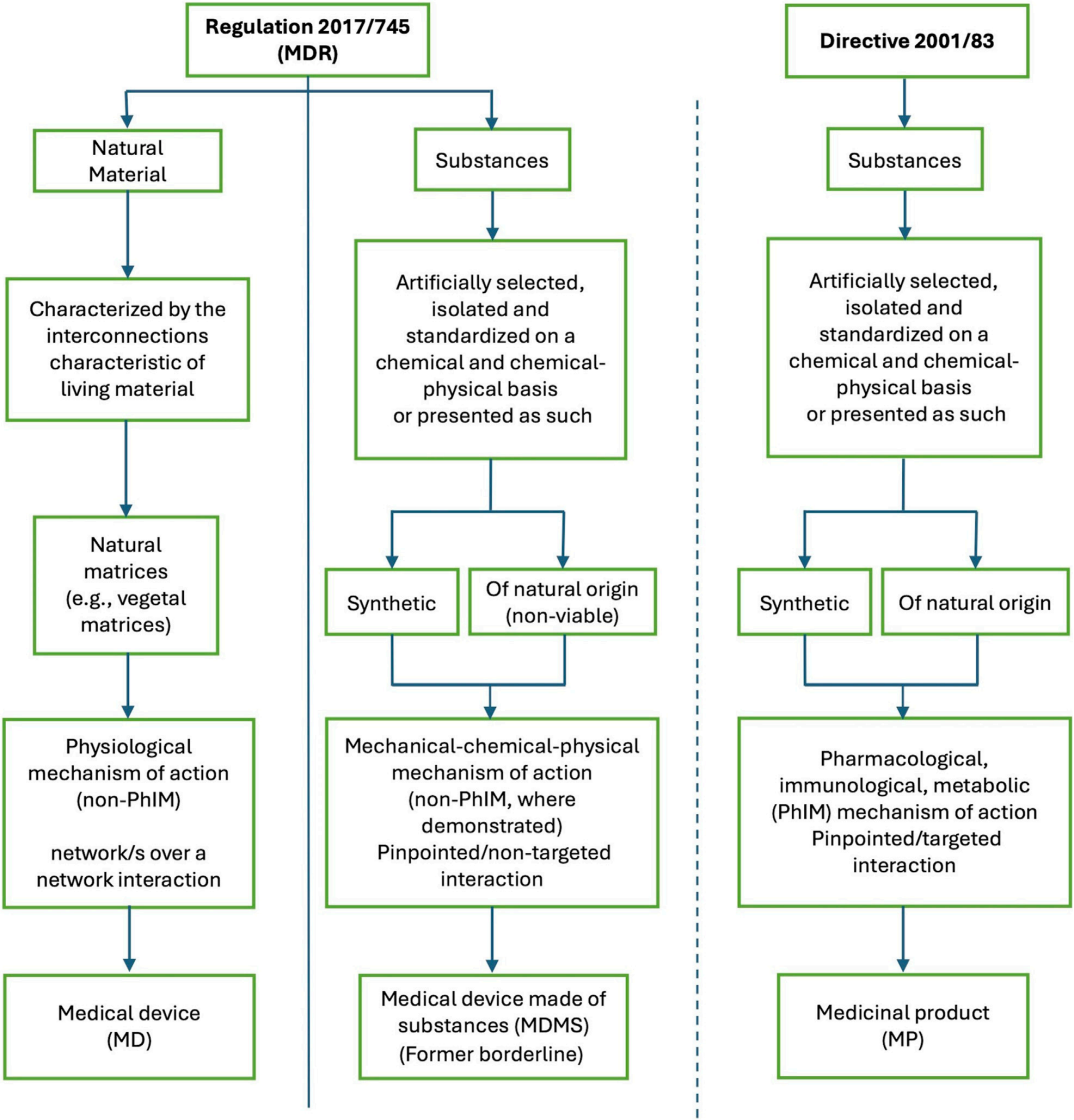
Regulatory pathway of a medical device, a medical device made of substances (also called substance based medical device), and a medicinal product, taking into account the main characteristics of each as identified by the certification/authorization requirements. The fundamental difference is whether the product is made of substances (either synthetic or of natural origin) or is constituted of natural material/s. The other differences, including the mechanism of action, are derived from this. Medical devices made of substances and medicinal products share the fundamental characteristic of being made from substances. Natural materials cannot be described as “substances or combinations of substances”, and are, therefore very different from both medical devices made of substances and medicinal products. MD: medical device; MDMS: medical device made of substances; MDR: Regulation 2017/745 Medical Device Regulation; MP: medicinal product; PhIM: pharmacological, immunological, metabolic.

On the other hand, the MDR seems well suited to regulate products that interact with the human body according to a “network/s over a network” model, without derogating from the guarantee of safety, efficacy and quality. This may seem an unconventional interpretation of the definition of a medical device, but the reality is that natural materials seem to fit neatly into this framework. Thus, MPs, MDMS and devices made of natural materials each have their own regulatory pathway (Figure 1).

Thus, MDR is the high-level regulatory framework that allows the enrichment of the therapeutic armamentarium with products consisting of natural materials, which should offer a new therapeutic approach based on restoring the physiological state and appear necessary to meet the many unmet medical needs that still exist today in areas such as oncology, orphan diseases, syndromes, functional and chronic degenerative diseases. It is therefore worthwhile, if not mandatory, to encourage the development of all products described by MDR. A proactive and constructive discussion on the possible and most appropriate regulatory framework for each type of therapeutic product is desirable.

## Abbreviations

Annex I: Annex I to Directive 2001/83, Directive: Directive 2001/83 on medicinal products, GSPR: General Safety and Performance Requirements, MDMS: medical devices made of substances (also called substance based medical devices), MDs: medical devices, MDR: Medical Device Regulation 2017/745, MoA: mechanism of action, MPs: medicinal products, “PhIM”: pharmacological, immunological, and metabolic.
